# Detection of dengue virus serotype 4 in Panama after 23 years without circulation

**DOI:** 10.3389/fcimb.2024.1467465

**Published:** 2024-10-01

**Authors:** María Chen-Germán, Dimelza Araúz, Celestino Aguilar, Melanie Vega, Claudia Gonzalez, Jessica Gondola, Lourdes Moreno, Lizbeth Cerezo, Leticia Franco, Jairo Mendez-Rico, Juan Miguel Pascale, Sandra López-Vergès, Alexander A. Martínez, Brechla Moreno

**Affiliations:** ^1^ Modular Specialized Laboratory, Department of Research in Virology and Biotechnology, Gorgas Memorial Institute for Health Studies, Panama City, Panama; ^2^ Department of Genomics and Proteomics, Gorgas Memorial Institute of Health Studies, Panama City, Panama; ^3^ Department of Microbiology and Immunology, University of Panama, Panama City, Panama; ^4^ National Department of Epidemiology, Ministry of Health, Panama City, Panama; ^5^ Infectious Hazard Management Unit, Health Emergencies Department, Pan American Health Organization, Washington, DC, United States; ^6^ Gorgas Memorial Institute of Health Studies, Panama City, Panama; ^7^ Virology Research Laboratory, Department of Research in Virology and Biotechnology, Gorgas Memorial Institute for Health Studies, Panama City, Panama

**Keywords:** dengue virus, Panama, arbovirus, surveillance, DENV-4

## Abstract

Panama is a country with endemic Dengue virus (DENV) transmission since its reintroduction in 1993. The four serotypes have circulated in the country and the region of the Americas, however, DENV-4 confirmed autochthonous cases have not been identified since 2000, despite its circulation in neighboring countries. Here, we report DENV-4 detection in Panama in the last four-month period of 2023 with co-circulation of the other serotypes, this was associated with a peak of dengue cases during the dry season even though most dengue outbreaks are described in the rainy season. Complete genomes of DENV-4 allowed us to determine that cases were caused by DENV-4 genotype IIb, the same genotype as 23 years ago, with high similarity to DENV-4 sequences circulating in Nicaragua and El Salvador during 2023. This report shows the importance of maintaining serotype and genotype surveillance for early detection of new variants circulating in the country.

## 1 Introduction

The first dengue-like epidemics reported in Panama date back to approximately 1699 (Brathwaite Dick et al., 2012). Official reports of dengue were made in 1904 and 1912 from the Panama Canal Zone (Carpenter and Sutton, 1905; Brathwaite Dick et al., 2012). Thanks to vector control campaigns, Panama had a period without dengue cases, until the decline of these programs, re-emergence of the vector and therefore re-introduction of dengue virus in 1993 (Quiroz et al., 1997), since then dengue virus (DENV) has been endemic in the country and has experienced the circulation of the four dengue serotypes; however, autochthonous cases of DENV-4 were only previously reported in 1998 and 2000. A single case of DENV-4 was detected in 2015, but it was an imported case from a traveler (Díaz et al., 2019).

Since 2010, dengue diagnostics in Panama have been decentralized. The diagnosis is based on the detection of NS1 antigen, molecular screening in specialized laboratories, and IgM detection. However, serotype surveillance is centralized at the Gorgas Memorial Institute for Health Studies (GMI), where a percentage of dengue-positive samples are received for molecular serotype surveillance. GMI also accepts suspected cases of dengue for diagnostics when health facilities cannot perform any dengue diagnostic tests. Additionally, a percentage of samples that meet sequencing selection criteria such as Cycle threshold (Ct.) value, location, travel history, and patient outcome are included for genotype surveillance for further characterization.

Dengue genomic surveillance allows the detection of the introduction or reintroduction of serotypes and genotypes, genotype replacement, or association of genotypes with new clinical manifestations or higher transmission. This study focused on the first cases of autochthonous DENV-4 detected in Panama through the National Surveillance System of Arbovirus after 23 years of no circulation between September 2023 and April 2024.

## 2 Materials and methods

### 2.1 Bioethical considerations

This study has been registered in the RESEGIS platform from DIGESA from the Ministry of Health in Panama under number 2652 and approved by the Bioethics Committee of Gorgas Memorial Institute for Health Studies N°071/CBI/ICGES/24.

### 2.2 National Surveillance System of Arbovirus

The national arboviral surveillance system of Panama consists of a network of laboratories with the capacity to perform NS1 antigen tests or viral detection through molecular assays in acute samples (≤5 days of symptom onset) and determination of IgM antibodies from convalescent samples (6-21 days from the onset of symptoms). The detection of NS1 and IgM antigens is carried out according to the availability of tests (immunochromatography, ELISA, FIA). Those facilities that do not have any of the mentioned methodologies send the samples for diagnosis to GMI.

The percentage established for serotype surveillance from national health facilities is 25% for positive acute samples for DENV and 10% for negative acute samples. The majority of Health Facilities perform NS1 tests, so the 10% of negative samples are re-tested for DENV, and other arboviruses like Chikungunya (CHIKV) and Zika (ZIKV) using a DENV-ZIKV-CHIKV multiplex Real Time PCR (Waggoner et al., 2016). Additionally, thanks to PAHO Laboratory networks like RELDA and VIGENDA, protocols for sequencing DENV are being implemented for genomic surveillance. For this purpose, 10% of positive samples will be selected randomly using a custom R script, considering national geographic areas with dengue cases that meet specific selection criteria like Ct. value up to 30 by qRT-PCR, and available sample volume for RNA extraction. Samples that have severe outcome, and/or travel history in the last 15 days, will also be considered for genotype surveillance.

### 2.3 Viral RNA extraction and whole genome sequencing

Viral RNA was extracted from human serum samples using MagMAX™ Viral/Pathogen II Nucleic Acid Isolation Kit (Cat.A48383, Thermo Fisher Scientific) in the KingFisher Flex. Samples were amplified using CDC DENV 1-4 real-time RT-PCR (Santiago et al., 2013). Of these samples those with Ct. values up to 30 were selected for sequencing using the CDC Next Generation Sequencing Protocol for DENV 1-4 Illumina MiSeq. This protocol was provided by the CDC and transferred to the laboratory by the Arbovirus Diagnosis Laboratory Network of the Americas (RELDA) facilitated by the Pan-American Health Organization (PAHO) VIGENDA program, based on tiled PCR. DENV-4 library preparation using the Nextera XT library prep kit (Illumina, San Diego, CA, USA) with dual indexes following the manufacturer’s instructions. Before pooling the samples for normalization, the libraries were quantified using a Qubit 2.0 Fluorometer. The sequencing was performed on an Illumina MiSeq platform at GMI using a V2 mid-output 500-cycle flow cell to obtain paired-end 250 bp reads from a 10 pM library with a 1% PhiX control.

### 2.4 Next-generation sequencing analysis

Consensus whole DENV-4 genomes were generated from the FASTQ files produced by Illumina MiSeq sequencing. Raw files were screened for quality and trimmed to remove primer sequence bias using Fastp (Chen et al., 2018). Cleaned FASTQ files were then aligned to a serotype-specific reference genome using the Burrows-Wheeler Alignment for short-reads (BWA-MEM) (Li and Durbin, 2009) along with the SAMtools view and sort packages (Li et al., 2009). The reference DENV-4 genome used was downloaded from GenBank Accession Number NC_002640. Finally, a consensus genome sequence in FASTA format was generated with Bcftools (Li, 2011). The genotype and serotype of the viral samples were confirmed from the generated consensus genomes using the DENV typing tool available from the Genome Detective resource (Vilsker et al., 2019), which uses phylogenetics and pairwise distance within and between groups to known references to assign a genotype to a sequence.

### 2.5 Phylogenetic analysis

Complete genomes of DENV-4 were downloaded from GenBank (**Supplementary Table 1A**) and the sequences were collated, and aligned using MAFFT v7.490 (Katoh and Standley, 2013). The best nucleotide substitution model was selected using jModelTest in IQ-TREE v2.2.0 (Minh et al., 2020). Maximum likelihood phylogenetic trees were inferred in IQ-TREE using the general unrestricted substitution model with invariable sites and 4 gamma categories (UNREST+FO+I+G4), and branch support was assessed by 1000 bootstrap iterations (Nguyen et al., 2015). Trees were visualized with FigTree v1.4.4 (available from http://tree.bio.ed.ac.uk/software/figtree).

Additionally, a subset was created extracting the gene E of DENV-4 using Geneious Prime v2024.0.4., to compare with older Panamanian sequences that circulated between the years 1998 and 2000 (Accession Numbers MH824741-MH824747) (**Supplementary Table 1B**).

## 3 Results

### 3.1 Epidemiological data

In 2023, Panama recorded the highest number of dengue cases in 30 years, with a total of 16,211 confirmed cases. Of these, 91% were classified as Dengue without warning signs (DNWS) and 9% as Dengue with warning signs (DWWS). Reports from the Ministry of Health in Panama documented a rise in cases from epidemiological week 44, reaching the peak incidence in week 49 (Cerezo and Moreno, 2024).

**Figure 1:**
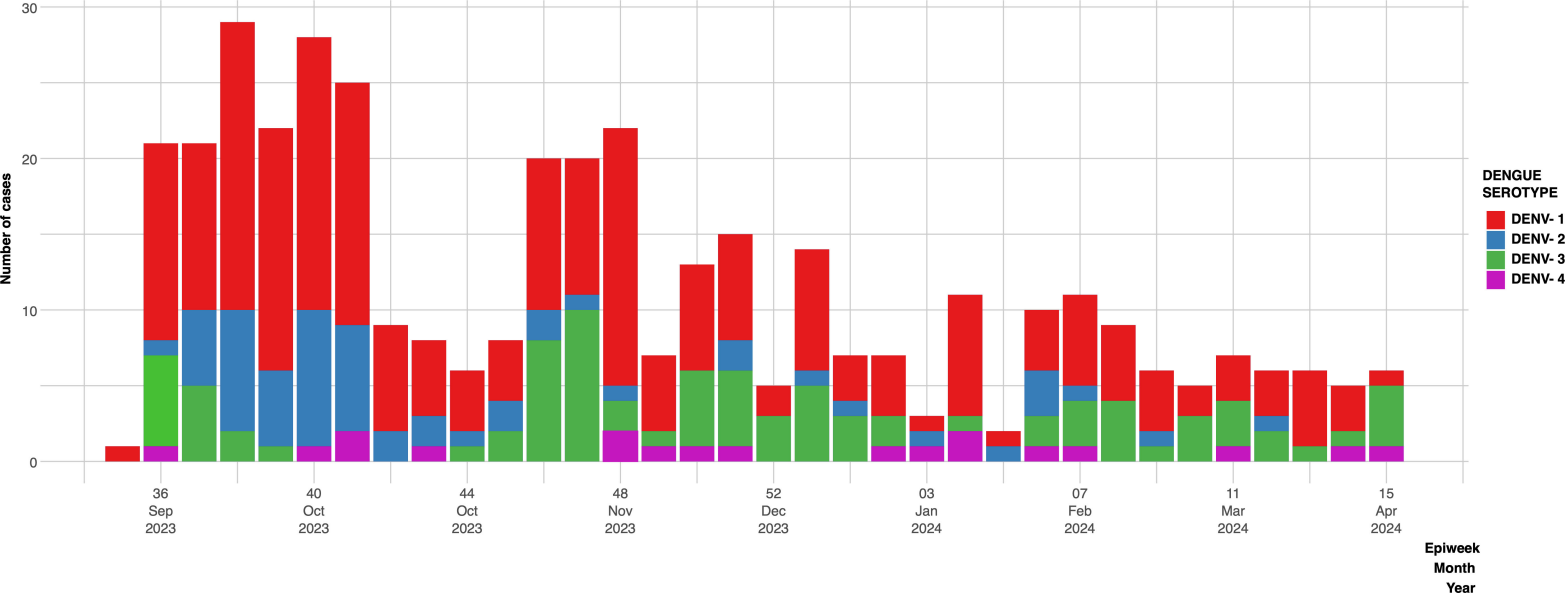
Epidemiological graph of serotype distribution of DENV positive cases characterized by qRT-PCR between September 2023 and April 2024. DENV-1(red bars), DENV-2(blue bars), DENV-3 (green bars), DENV-4 (purple bars).

This study was conducted between epidemiological week 36 of 2023 (September) and week 16 of 2024 (April). During this period, GMI received 780 samples for DENV screening. The percentage of positive samples for DENV was 53% out of this, 4.4% was attributed to DENV-4 (**Figure 1**), this was the first time since 2000 that autochthonous cases of DENV-4 were detected in co-circulation with the other serotypes DENV-1 (58.8%), DENV-2 (15.6%), DENV-3 (21.2%).

**Figure 2:**
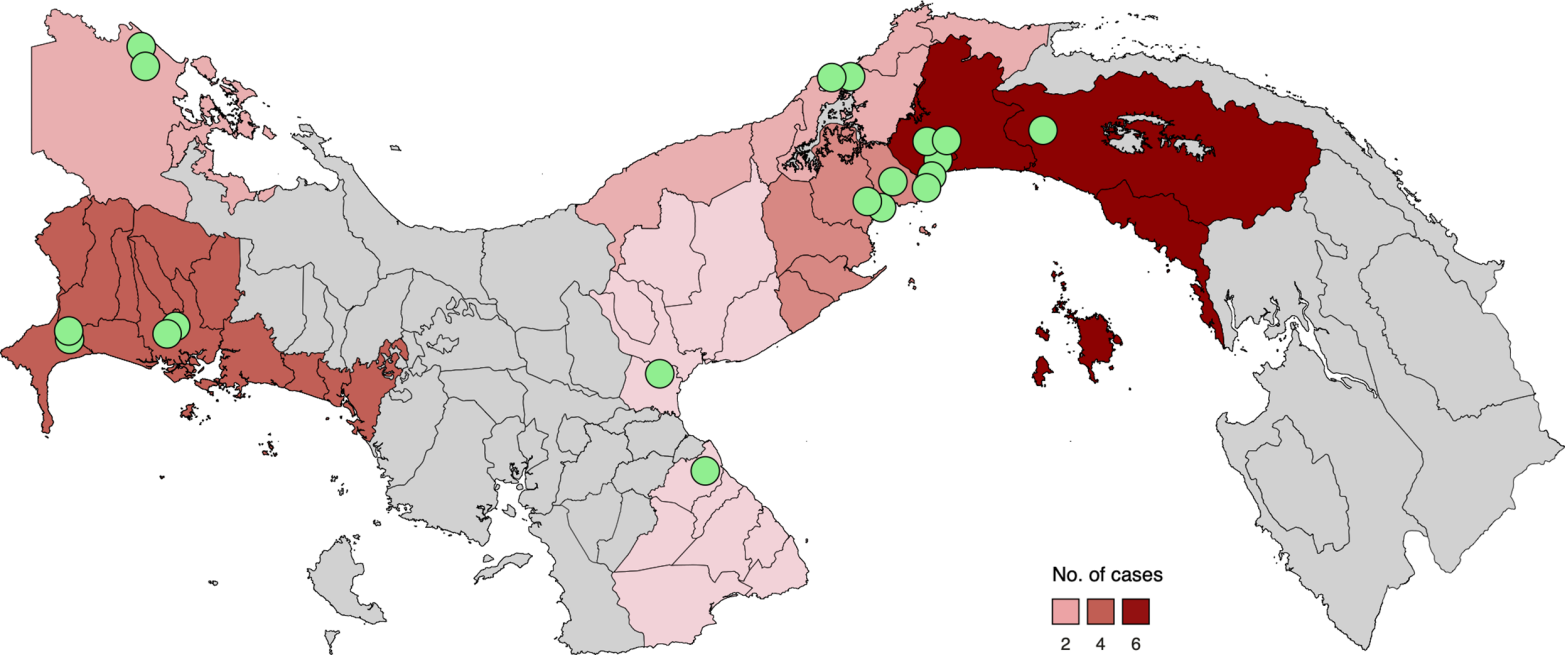
Map representing the number of DENV-4 confirmed cases per province. Provinces with DENV-4 cases are represented with a red grade of colors depending on the number of confirmed cases, whereas dots indicate the location of DENV-4 positive samples collection.

Panama is divided into ten provinces and five provincial-level autochthonous Indigenous regions. The provinces with positive serotype 4 cases (DENV-4) were Panama (the Metropolitan area of Panama city), Panamá Oeste, Chiriquí, Bocas del Toro, Coclé, Colón, and Los Santos (**Figure 2**). The metropolitan area of Panama had the highest number of reported cases, followed by Chiriquí. The initial case of DENV-4 detected through the system was in October of 2023, specifically in week 41 in a sample from Coclé, which is approximately a 2-hour drive from the city. However, locations that are farther from GMI, like Chiriquí and Bocas del Toro, face limitations for periodic shipments, usually preserving samples at -80°C and making the shipment when transportation is available. Taking this into account, at the beginning of February 2024, GMI received a group of samples from Bocas del Toro, a very touristy province and one of the farthest from the GMI. These samples were from the last 4 months of 2023; among them, we detected a case of DENV-4 that epidemiologically corresponded 5 weeks before our first detection, with symptoms starting on EW 36. The same scenery repeated with a group of samples from Chiriquí when GMI received a group of samples from the last 3 months of 2023 in January of 2024, and DENV-4 was detected in a sample from EW 40, one week before the initial detection of this serotype.

Epidemiological data from these DENV-4 samples reveals that the most common symptoms among these patients were fever, chills, headache, myalgia, and retro-orbital pain. Less common symptoms included arthralgia, exanthema, diarrhea, nausea, abdominal pain, and sore throat (**Supplementary Table 2**). No hospitalized or fatal cases were associated with DENV-4 as the clinical classification of all DENV-4 cases presented in this study was Dengue without warning sign (DWWS).

### 3.2 Phylogenetic analysis and mutations

**Figure 3:**
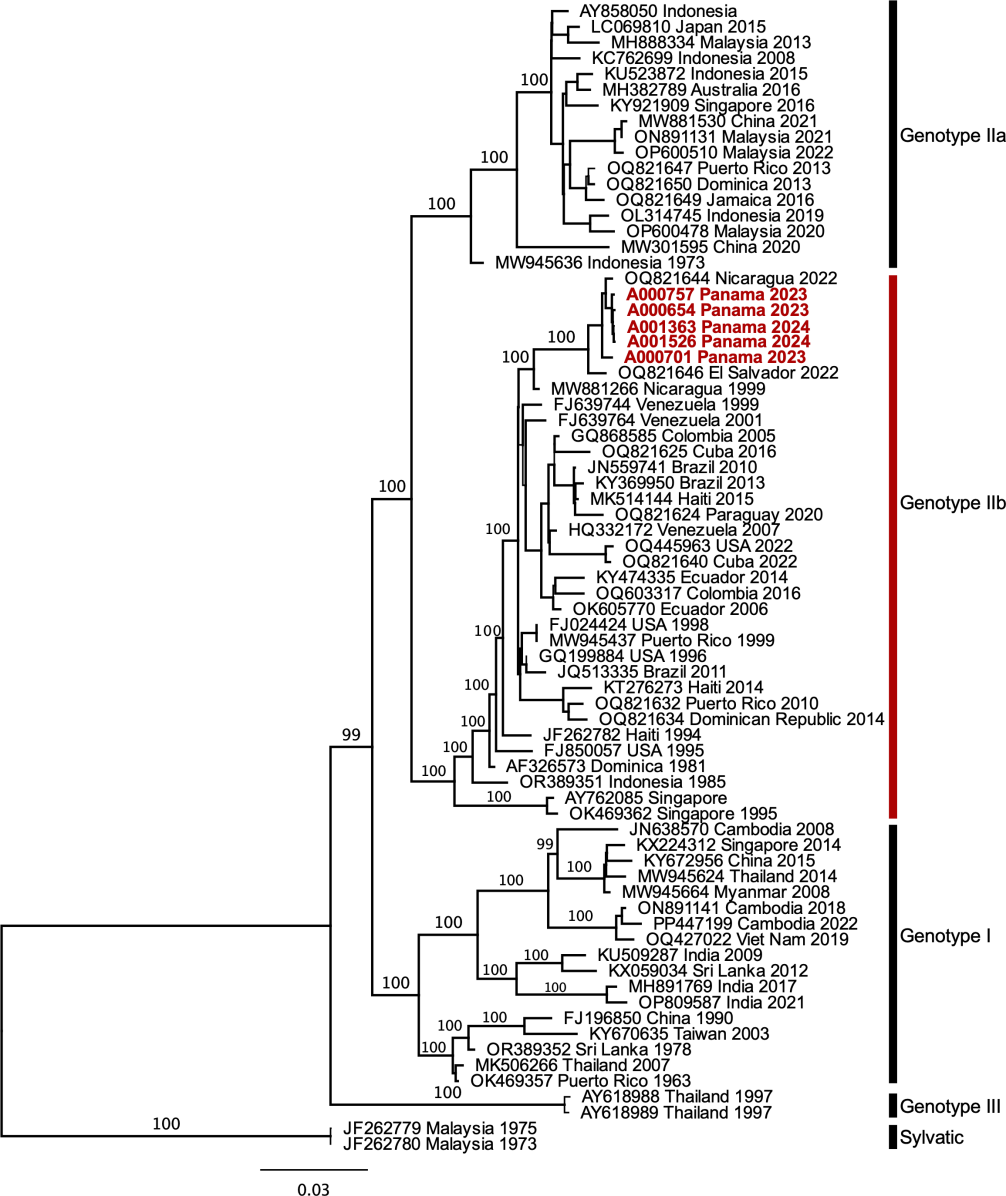
Maximum likelihood phylogeny of DENV-4 based on complete genome sequences. The phylogenetic tree was constructed based on the model UNREST+FO+I+G4, with 1000 bootstrap replicates. The DENV-4 sequences obtained from Panama in this study are highlighted in red. Genotype names were marked accordingly. IQ-TREE maximum likelihood bootstrap support values are indicated above the branches. All internal nodes have bootstrap values of >90.

Of the 19 DENV-4 positive samples detected through the National Arboviral Surveillance System, we were able to sequence 5 samples, and all the sequences obtained were classified as DENV-4 genotype IIb. The analysis of DENV-4 complete genome sequences shows that Panamanian sequences from 2023 and 2024 formed a monophyletic clade with those from Nicaragua and El Salvador from 2022 (**Figure 3**).

**Figure 4:**
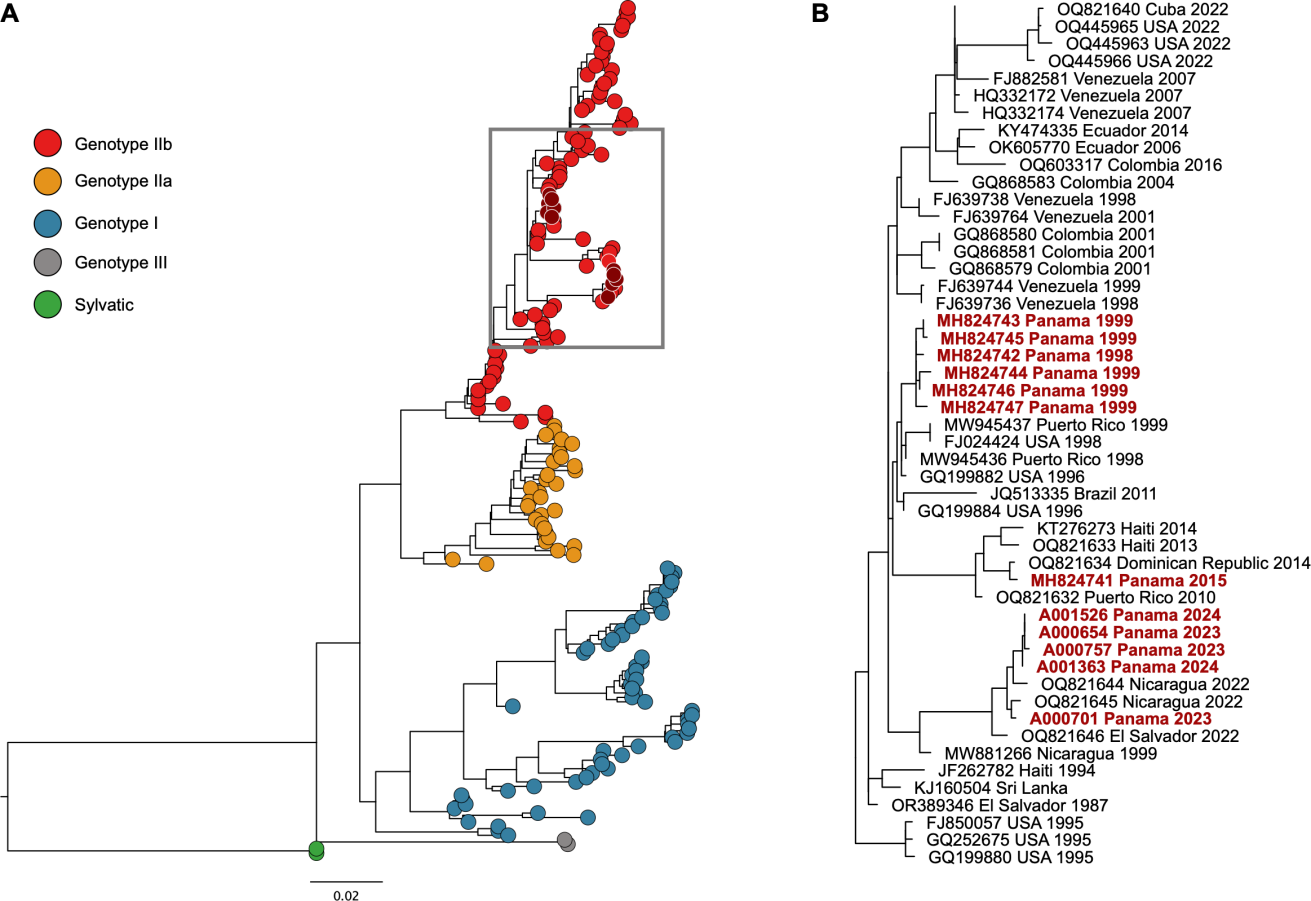
Phylogenetic analysis of the E gene of DENV-4 from Panama. **(A)** The maximum likelihood tree was constructed on a 1541 bp fragment of the E gene under the GTR+F+I+G4 substitution model and 1000 bootstrap resampling. The DENV-4 sequences obtained from Panama are highlighted in dark red solid dots. Genotypes of DENV-4 are shown in red, yellow, blue, gray, and green solid dots. **(B)** Phylogeny of a subset of DENV-4 genotype IIb isolates. Isolates from Panama are in red font. Unless otherwise indicated, all nodes had more than 75% support of bootstrap. Scale bars indicate nucleotide substitutions per site.

The five nearly complete genomes were almost identical across the whole genome and the E gene, with sequence identity above 99.41% and 99.68%, respectively. Notably, the sequence identity between the five E gene fragments from the genomes reported in the present study and previously published Panamanian DENV-4 E gene nt sequences showed lower identities, between 95.81 and 97.50%. Thus, even if they are all part of the DENV-4 genotype IIb, Panamanian DENV-4 E gene sequences from 2023 and 2024 samples are in a different clade than E gene sequences from Panamanian DENV-4 from 1998 and 1999 and the imported case from 2015 (**Figure 4**).

## 4 Discussion

Here we demonstrate how national molecular serotype surveillance of dengue facilitated the identification of DENV-4, underscoring the critical role of this surveillance program. The detection of DENV-4 occurred with simultaneous circulation of the other 3 serotypes (DENV-1, DENV-2, and DENV-3) having the 4 serotypes at the same time, leaving us in an epidemiological scenario that hasn’t been seen since 2000.

The genetic characterization of a group of DENV-4 acute samples showed that recent viruses are part of genotype IIb and are highly similar to viruses circulating one year before in Central America.

DENV-4 was detected in EW 36 when there were already three other serotypes co-circulating in the country followed by a rise in cases after EW 44. In 2023, Panama reported 17 deaths, although we cannot attribute the registered deaths to this serotype, according to the epidemiological data from the Ministry of Health, the largest number of deaths due to dengue occurred from EW38 (Cerezo and Moreno, 2024). Previous studies suggested that the introduction of new serotypes could be associated with an increase of dengue cases, outbreaks, and more importantly with higher severity (OhAinle et al., 2011; Gowri Sankar et al., 2021).

The percentage of DENV-4 cases among the molecular positive samples was low, indicating that it’s not the predominant serotype, and is not replacing others. Clinically all these cases were classified as Dengue without warning sign (DWWS), suggesting that it was not the main serotype associated with disease severity. However, as the number of samples received at GMI per month from each province for molecular surveillance was low, they were probably not representative of the transmission pattern of the four serotypes in the country, making it difficult to conclude which was the exact role of DENV-4 detection and circulation versus the other three serotypes in this period. It has been described that some serotypes are associated with a higher risk of severe dengue for primary infections or for secondary infections after other specific serotypes (Narvaez et al., 2024). The information regarding dengue primary or secondary dengue infections was not available for the acute samples received, therefore, it remains challenging to determine whether DENV-4 infections in Panama could present differently during primary infection compared to other circulating serotypes, or if secondary DENV-4 infection following other serotypes are associated with severity. This underscores the importance of establishing a sustainable and representative molecular serotype surveillance program, along with enhanced data sharing among all surveillance components (clinical, epidemiology, laboratory). Such efforts are crucial for gaining a better understanding of DENV serotypes transmission dynamics and their impact during outbreaks.

With the epidemiological and molecular information available for this study, we can hypothesize that DENV-4 introduction was through Bocas del Toro (EW 36) and Chiriquí (EW 40), which are the provinces that share borders with Costa Rica and then spread through the country. At the time of the first Panamanian DENV-4 case with initial symptoms onset in EW 36, Costa Rica already had the circulation of the four dengue serotypes since they reported the first re-introduction of DENV-4 in EW 38 of 2022, almost a year before our cases (González, 2023), like other Central American countries. Our phylogenetic data shows that Panamanian sequences are closely related to Nicaragua and El Salvador sequences. Nicaragua also reported the re-introduction of DENV-4 in 2022 and shared similarities with El Salvador sequences (Cerpas et al., 2024). As no sequences were available from Costa Rican DENV-4 cases or other Central American countries at the time of our analysis, it is difficult to determine the origin of our DENV-4 cases and to perform phylogeographic analysis for spatial diffusion studies between countries.

Serotype and genomic information accompanied by epidemiological data is fundamental to understanding epidemic, endemic, or newly introduced serotypes and/or genotypes, and their impact on dengue epidemiology of dengue disease severity (Wallau et al., 2023). Genomic surveillance enables us to track the historical evolution of viruses in specific locations and understand their transmission pathways, identifying the movement patterns of the virus and potential sources of outbreaks (Brito et al., 2021; Branda et al., 2023; Tosta et al., 2023). Identifying the location of cases helps us to focus and prioritize efforts in high-risk areas through public health campaigns for awareness, reducing mosquito breeding sites, and implementing vector control policies (Pang et al., 2017). First dengue sequences began focusing mainly on the E protein (Santiago et al., 2019), however, changes in other parts of the genome could be occurring and not been detected because of the approach (Ladner et al., 2014). Now complete genome sequencing provides reliable and affordable genetic information that can be considered in the design, implementation, and effectiveness of vaccine candidates, in- vivo and *in-vitro* experiments, and mutations that could increase severity or transmission (Rico-Hesse, 2003; Pang et al., 2017). Thus, it is important to strengthen the surveillance programs at regional and national level to have a better understanding of DENV transmission in the Americas.

## Data Availability

The datasets presented in this study can be found in online repositories. The names of the repository/repositories and accession number(s) can be found below: https://www.ncbi.nlm.nih.gov/genbank/, PQ014892 https://www.ncbi.nlm.nih.gov/genbank/, PQ014893 https://www.ncbi.nlm.nih.gov/genbank/, PQ014894 https://www.ncbi.nlm.nih.gov/genbank/, PQ014895 https://www.ncbi.nlm.nih.gov/genbank/, PQ014896.
