# Supplementary material for: Detection of dengue virus serotype 4 in Panama after 23 years without circulation
Source: Front Cell Infect Microbiol. 2024 Oct 1;14:1467465. doi: 10.3389/fcimb.2024.1467465 (PMC11473613; doi:10.3389/fcimb.2024.1467465)
Supplement: Supplementary file 1 [file DataSheet1.pdf]

frontiers

Supplementary Material

***1.Supplementary Table 1A**: GenBank Accession numbers of sequences used for complete genome phylogenetic analysis*

Accession Number	Country	Year
**MH382789**	Australia	2016
**JN559741**	Brazil	2010
**JQ513335**	Brazil	2011
**KY369950**	Brazil	2013
**JN638570**	Cambodia	2008
**ON891141**	Cambodia	2018
**PP447199**	Cambodia	2022
**FJ196850**	China	1990
**KY672956**	China	2015
**MW301595**	China	2020
**MW881530**	China	2021
**GQ868585**	Colombia	2005
**OQ603317**	Colombia	2016
**OQ821625**	Cuba	2016
**OQ821640**	Cuba	2022
**AF326573**	Dominica	1981
**OQ821650**	Dominica	2013
**OQ821634**	Dominican Republic	2014
**KY474335**	Ecuador	2014
**OK605770**	Ecuador	2006
**OQ821646**	El Salvador	2022
**JF262782**	Haiti	1994
**KT276273**	Haiti	2014
**MK514144**	Haiti	2015
**KU509287**	India	2009
**MH891769**	India	2017
**OP809587**	India	2021
**AY858050**	Indonesia	
**KC762699**	Indonesia	2008
**KU523872**	Indonesia	2015
**MW945636**	Indonesia	1973
**OL314745**	Indonesia	2019
**OR389351**	Indonesia	1985
**OQ821649**	Jamaica	2016
**LC069810**	Japan	2015
**JF262779**	Malaysia	1975
**MH888334**	Malaysia	2013

Accession Number	Country	Year
**ON891131**	Malaysia	2021
**OP600478**	Malaysia	2020
**OP600510**	Malaysia	2022
**JF262780**	Malaysia	1973
**MW945664**	Myanmar	2008
**MW881266**	Nicaragua	1999
**OQ821644**	Nicaragua	2022
**PQ014892**	Panama	2023
**PQ014893**	Panama	2023
**PQ014894**	Panama	2024
**PQ014895**	Panama	2024
**PQ014896**	Panama	2023
**OQ821624**	Paraguay	2020
**MW945437**	Puerto Rico	1999
**OK469357**	Puerto Rico	1963
**OQ821632**	Puerto Rico	2010
**OQ821647**	Puerto Rico	2013
**AY762085**	Singapore	
**KX224312**	Singapore	2014
**KY921909**	Singapore	2016
**OK469362**	Singapore	1995
**KX059034**	Sri Lanka	2012
**OR389352**	Sri Lanka	1978
**KY670635**	Taiwan	2003
**AY618988**	Thailand	1997
**AY618989**	Thailand	1997
**MK506266**	Thailand	2007
**MW945624**	Thailand	2014
**FJ024424**	USA	1998
**FJ850057**	USA	1995
**GQ199884**	USA	1996
**OQ445963**	USA	2022
**FJ639744**	Venezuela	1999
**FJ639764**	Venezuela	2001
**HQ332172**	Venezuela	2007
**OQ427022**	Viet Nam	2019

Supplementary Table 1B: *GenBank Accession numbers of sequences added used for E gene phylogenetic analysis*

Panamanian Sequences Added for Subset analysis		
**Accession Number**	**Country**	**Year**
**MH84742**	Panamá	1998
**MH84743**	Panamá	1999
**MH84744**	Panamá	1999
**MH84745**	Panamá	1999
**MH84746**	Panamá	1999
**MH84747**	Panamá	1999
**MH84748**	Panamá	2015

2. ***Supplementary Table 2****: Heat map of symptoms and signs of the 19 detected DENV-4 patients.*

Symptoms	n=1<br>0-5 years	n=3<br>6-17 years	n=7<br>18-35 years	n=7<br>36-59 years	n=1<br>> 60 years	n=19<br>over all
**Fever**	100	100	100	100	100	100
**Chill**	100	67	71	86	100	85
**Headache**	0	67	100	86	0	50
**Retroorbital pain**	100	33	71	57	0	52
**Myalgia**	100	67	57	86	0	62
**Arthralgia**	0	0	43	57	100	40
**Exanthema**	100	0	29	29	0	31
**Sore throat**	0	0	0	14	0	3
**Diarrhea**	100	33	43	29	0	41
**Nausea**	0	67	29	0	0	19
**Gum bleading**	0	0	14	0	0	3
**Abdominal pain**	0	33	14	0	0	10
